# Prenatal influences on postnatal neuroplasticity: Integrating DOHaD and sensitive/critical period frameworks to understand biological embedding in early development

**DOI:** 10.1111/infa.12588

**Published:** 2024-03-06

**Authors:** Emma T. Margolis, Laurel J. Gabard‐Durnam

**Affiliations:** ^1^ Department of Psychology Northeastern University Boston Massachusetts USA

## Abstract

Early environments can have significant and lasting effects on brain, body, and behavior across the lifecourse. Here, we address current research efforts to understand how experiences impact neurodevelopment with a new perspective integrating two well‐known conceptual frameworks – the Developmental Origins of Health and Disease (DOHaD) and sensitive/critical period frameworks. Specifically, we consider how prenatal experiences characterized in the DOHaD model impact two key neurobiological mechanisms of sensitive/critical periods for adapting to and learning from the postnatal environment. We draw from both animal and human research to summarize the current state of knowledge on how particular prenatal substance exposures (psychoactive substances and heavy metals) and nutritional profiles (protein‐energy malnutrition and iron deficiency) each differentially impact brain circuits' excitation/GABAergic inhibition balance and myelination. Finally, we highlight new research directions that emerge from this integrated framework, including testing how prenatal environments alter sensitive/critical period timing and learning and identifying potential promotional/buffering prenatal exposures to impact postnatal sensitive/critical periods. We hope this integrative framework considering prenatal influences on postnatal neuroplasticity will stimulate new research to understand how early environments have lasting consequences on our brains, behavior, and health.

## 1 INTRODUCTION

Early environments can have significant and lasting effects on brain, body, and behavior over the lifecourse (e.g., Berens et al., 2017; Marín, 2016; Nelson & Gabard‐Durnam, 2020; Reh et al., 2020; Van den Bergh, 2011; Werker & Hensch, 2015). At the most general level, biological embedding describes the processes by which pre‐ and postnatal environmental factors “get under the skin” to have these persistent effects on structure, physiology, and function (Aristizabal et al., 2020; Champagne, 2018; Hertzman, 2012; Nelson, 2013; Rutter, 2016; Shonkoff et al., 2009; Traniello & Robinson, 2021). Biological embedding comprises both opportunities and vulnerabilities for the developing system: these processes allow individuals to learn and adapt to their specific environmental conditions, but adverse early conditions or subsequent deviations from learned environments can render individuals at risk for maladaptive or clinical outcomes (Bateson et al., 2014; Berens et al., 2017; Gluckman & Hanson, 2004; Nelson & Gabard‐Durnam, 2020).

Multiple conceptual frameworks have emerged to describe the mechanisms by which the environment becomes biologically embedded with persistent consequences across research areas and levels of analysis, ranging from genetics to neuroscience to epidemiology (Bateson et al., 2014; Camerota & Willoughby, 2021; Knudsen, 2004; Van den Bergh, 2011). Importantly, though biological embedding mechanisms operate and interact across these levels of analysis to enact their complex effects over development, the corresponding conceptual frameworks have largely matured through separate, parallel literatures (though see Bock et al., 2015; Camerota & Willoughby, 2021; Colombo et al., 2019; Hartman & Belsky, 2021; Ozen et al., 2023). Thus, explicit discussions of interactions and integrations between framework mechanisms are needed to deepen understanding of these complex biological embedding phenomena. To address this need, we propose integrating two prominent conceptual frameworks of biological embedding – the Developmental Origins of Health and Disease (DOHaD) framework and the neural sensitive/critical periods framework.

The DOHaD framework of biological embedding emerged with a focus on how prenatal experiences change prenatal physiology and function with consequences for postnatal development and health outcomes into adulthood, especially with respect to the emergence of non‐communicable diseases and physical health conditions (Barker et al., 1989, 2002; Eriksson, 2016; Eriksson et al., 2002; Hanson, 2015; Jazwiec & Sloboda, 2019; Leguizamon, 2022; Silveira et al., 2007; Uauy et al., 2011; Wadhwa et al., 2009). Extensive research within the DOHaD framework has linked prenatal environmental conditions such as nutritional profiles (e.g., protein‐energy malnutrition ‐ Ozanne & Hales, 2004; Remacle et al., 2011; iron deficiency ‐ Barks et al., 2019; Doom & Georgieff, 2014) and substance exposures (e.g., alcohol ‐ Asiedu et al., 2021; Lunde et al., 2016; nicotine ‐ Aagaard‐Tillery et al., 2008) to fetal and early postnatal phenotypes (e.g., birthweight ‐ Aagaard‐Tillery et al., 2008; Tellapragada et al., 2016). These early postnatal phenotypes are then associated with subsequent adult health conditions (e.g., cardiovascular or metabolic disease; Alves & Alves, 2023; Asiedu et al., 2021; Barker et al., 2002; De Boo & Harding, 2006; Gluckman & Hanson, 2004; Hanson, 2019; Lunde et al., 2016; Remacle et al., 2011; Silveira et al., 2007) to identify the lasting effects of those prenatal environmental conditions within the DOHaD framework. Postnatal environmental conditions also play a role within the DOHaD framework, in that prenatal changes and adaptations in response to experiences in utero may lead to especially poor health outcomes when the postnatal environmental cues do not match the prenatal environmental cues (Bateson et al., 2014; Gluckman & Hanson, 2004). However, there has been less focus on postnatal plasticity and adaptation within the DOHaD framework, especially with respect to the brain and mental health (though see Monk & Fernández, 2022; Van den Bergh, 2011).

Conversely, the neural sensitive/critical period framework offers biological embedding mechanisms that have traditionally focused on peri‐ and early postnatal windows of elevated neuroplasticity and adaptation to the environment, termed sensitive/critical periods, that have lasting impacts on physiology and function through adulthood (Gabard‐Durnam & McLaughlin, 2020; Hensch, 2005; Knudsen, 2004). Evidence for peri‐ and postnatal neural sensitive/critical periods supporting learning exists across a variety of domains from vision to language and attachment/imprinting, making these biological embedding mechanisms key to healthy neurodevelopment (see Hensch, 2005; Opendak et al., 2017; Upton & Sullivan, 2010; Werker & Hensch, 2015). Of course the brain, including the neural substrates of sensitive/critical periods, undergoes extensive development prenatally (Jakovcevski et al., 2009; Marín‐Padilla, 2011; O’Rahilly & Müller, 2006). However, the neural sensitive/critical period framework has had limited consideration of the prenatal origins and environmental influences on these biological embedding mechanisms that unfold peri‐ and postnatally (though see Linderkamp & Linderkamp‐Skoruppa, 2021).

Here we argue that integrating the DOHaD and sensitive/critical period biological embedding frameworks will provide new opportunities to understand early brain development. Specifically, we articulate how the prenatal environmental factors that have robust effects within the DOHaD framework also impact how postnatal sensitive/critical period processes unfold to shape neurodevelopment. That is, not only do prenatal experiences impact physiological adaptation prenatally, they also impact mechanisms for physiological adaptation postnatally (i.e., prenatal influence on postnatal neuroplasticity). This integrated framework provides new insights relative to the DOHaD and sensitive/critical period frameworks independently by considering postnatal neuroplasticity and adaptation in the context of DOHaD concepts and prenatal environmental influences in the context of sensitive/critical period concepts, respectively. Our integrated framework also differs from biological embedding models involving repeated adversity exposures like multiple‐hit (Daskalakis et al., 2013; Meaney, 2001), cumulative (Evans et al., 2013; Evans & Kim, 2007), or sensitization frameworks (Hammen et al., 2000; Harkness et al., 2006; Stroud, 2019) by considering multiple downstream consequences of a single prenatal exposure to either adverse or promotional environmental factors (though our framework may integrate further with these multiple‐adversity models by elaborating the consequences of an initial adverse experience where subsequent adversity is also experienced). Finally, our integrated framework's focus on how prenatal environments influence postnatal neuroplasticity is distinct but complements the differential‐susceptibility framework's focus on prenatal adversity's impacts on postnatal susceptibility to environmental variability (e.g., Pluess & Belsky, 2011). In these ways, we believe our integration of DOHaD and sensitive/critical period frameworks provides a new perspective on biological embedding phenomena.

We elaborate our integrated framework and demonstrate its empirical utility in the following ways. First, we characterize the prenatal environmental factors within the DOHaD framework that we consider within our integrated framework, focusing on substance exposures and prenatal nutrition as categories of these *in utero* influences with rich literature across species and which produce significant public health burden. Specifically, we focus on psychoactive substances (i.e., alcohol, nicotine, cocaine, cannabis) and environmental pollutants (i.e., heavy metals – lead, cadmium, mercury, arsenic) as examples of prenatal substance exposures, and we focus on protein‐energy malnutrition and iron deficiency as examples of prenatal nutritional profiles. Next, we define sensitive/critical period concepts and their neurobiological substrates. We then demonstrate the utility of this integrated framework by providing empirical examples across species for how each of the DOHaD‐characterized classes of prenatal experiences impacts two key neurobiological substrates of sensitive/critical periods postnatally. Lastly, we highlight research priorities that our integrated framework provides to drive new insight into how prenatal environments exert such striking effects on postnatal development. We hope this integrated framework thus facilitates new ways to understand and research biological embedding of environments in early life.

## 2 DOHaD AND PRENATAL ENVIRONMENTAL INFLUENCES

The DOHaD framework has facilitated a rich empirical literature on the nature and mechanisms of prenatal environmental factors that most robustly impact prenatal physiology, metabolism, and function with lasting consequences postnatally. Two such classes of prenatal environmental factors that have emerged as particularly robust influences on fetal and postnatal phenotypes include prenatal substance exposures and nutritional profiles.

### 2.1 Prenatal substance exposures

Prenatal substance exposures researched within the DOHaD framework include both psychoactive substances such as alcohol, nicotine, cocaine, or cannabis as well as environmental pollutants such as heavy metals. With respect to prenatal psychoactive substance exposures (e.g., alcohol, nicotine, cocaine, cannabis), each of these substances has received extensive empirical focus across levels of analysis on their fetal and downstream impacts (De Genna et al., 2022; Eiden et al., 2023; Ernst et al., 2001; Flak et al., 2014; Jacobson et al., 2021; Lester & Padbury, 2009; Pini et al., 2024). Moreover, as the psychoactive descriptor implies, they are all known to directly impact the brain. Thus, these substances are robust candidates to assess prenatal impacts on neural sensitive/critical period phenomena in our integrated framework.

We also focus on these substances because they continue to pose a significant global public health burden (Tavella et al., 2020; World Health Organization, 2014). For example, a 2020 report estimates that 8%–11% of pregnant women in the United States endorsed use of alcohol, tobacco, or illicit drugs (United States Department of Health and Human Services, 2022), which is a notable recent increase in prevalence from prior estimates (e.g., 5.2%) of endorsement by pregnant women in 2006 and 2007 (United States Department of Health and Human Services, 2008). Globally, a recent meta‐analysis of endorsement of illicit drugs during pregnancy found a prevalence of 12.28% for studies using toxicological analysis (Tavella et al., 2020). In this meta‐analysis, cannabis and cocaine were the most commonly endorsed substances during pregnancy (Tavella et al., 2020). For all of these reasons, our integrated framework focuses specifically on prenatal influences of alcohol, nicotine, cannabis, and cocaine as key prenatal psychoactive substance exposures.

Environmental pollutants are the second type of substance exposure that has been well‐characterized within the DOHaD framework that we assess in our integrated framework. There are many kinds of environmental pollutants including various chemicals, plastics, and heavy metals. Here, we focus on four heavy metals pollutants identified by the World Health Organization recently as chemicals of public health concern: lead, cadmium, mercury, and arsenic (World Health Organization, 2020a). Moreover, these four metals are well‐defined entities relative to other environmental pollutants (e.g., plastics include a large number of substances within that category that may not have uniform impacts). In addition, each of these heavy metals can cross the placental barrier to impact fetal neurodevelopment (Caserta et al., 2013; Iwai‐Shimada et al., 2019; Jin et al., 2006), as required for assessment in our integrated framework, with some of these metals accumulating in the fetus in far larger dose‐weight ratios than at later life stages (Dack et al., 2021).

We also focus on these four heavy metals because as their World Health Organization categorization suggests, they are both prevalent and pose a global public health concern. Specifically, the WHO recently estimated in 2019 that around 30% of intellectual disability worldwide can be attributed to lead exposure (which can occur via contaminated paint, drinking water, air, etc.; World Health Organization, 2021a). Despite these staggering statistics, as of March 2023, only 48% of countries have laws regulating lead paint (World Health Organization, 2021a), making it likely that such exposures will continue to be a public health issue. Cadmium exposure can occur via diet and contaminated drinking water, and studies have identified unsafe levels of cadmium in drinking water from a variety of countries across Asia, Africa, and Europe (e.g., Pakistan, Nigeria, Saudi Arabia, the Netherlands, and Sweden; Rehman et al., 2018; World Health Organization, 2019b). Moreover, mercury exposure can occur from a variety of common sources (Basu et al., 2018; United States Environmental Protection Agency, 2015; World Health Organization, 2010, 2021b), including diet (e.g., consumption of seafood), industrial processes (e.g., proximity to power stations, residential heating systems, waste incinerators, mining), and even cosmetic use. Finally, arsenic is also a prevalent contaminant, as the WHO estimated in 2002 that at least 140 million people in at least 50 countries were exposed to unsafe levels of arsenic via drinking water (World Health Organization, 2019a), and a 2020 study estimated as many as 220 million people may now be exposed worlwide (Podgorski & Berg, 2020). Given these reasons, our integrated framework highlights lead, cadmium, mercury, and arsenic as key prenatal heavy metal substance exposures.

### 2.2 Nutritional profiles

A second class of prenatal environmental factors that has emerged within DOHaD as particularly impactful on fetal and postnatal phenotypes is the prenatal nutritional profile (Jazwiec & Sloboda, 2019; Ozanne & Hales, 2004; Remacle et al., 2011; Uauy et al., 2011). For several reasons, we focus specifically on protein‐energy malnutrition and iron‐deficiency factors. Protein‐energy malnutrition/undernutrition refers to insufficient intake or absorption of protein (and in more severe cases, overall caloric intake) to meet metabolic demands and may occur in situations of food insecurity or scarcity (Batool et al., 2015; Grover & Ee, 2009; Martin & Dombrowski, 2008; World Health Organization, 2000). Prenatal protein‐energy malnutrition has wide‐ranging neurodevelopmental consequences, making it a strong candidate to assess in our integrated neurodevelopmental framework (Batool et al., 2015; Lassi et al., 2022). Moreover, protein‐energy malnutrition continues to pose a global health burden. An estimated 6.9 million pregnant and breastfeeding women were acutely malnourished in 2022 in the 12 countries with the most extreme food and nutrition crises, a 25% increase from 2020 (United Nations Children's Fund, 2023). In a survey of 10 countries in Africa, anywhere from 19.5% to 23.5% of pregnant women experience protein‐energy malnutrition during pregnancy (Desyibelew & Dadi, 2019). Moreover, globally, overall food insecurity during pregnancy (which can result in protein‐energy malnutrition) ranges from 9% to 87.9%, with majority world countries often experiencing higher rates of food insecurity (Ramalho et al., 2017).

We also focus on prenatal iron deficiency as the second key nutritional factor to consider within the DOHaD framework. A rich literature has established that iron is critical for brain development (Reid & Georgieff, 2023). However, high iron requirements during pregnancy as well as increased maternal blood volume pose a risk for iron‐deficiency, iron deficiency anemia, and iron‐deficiency in the brain (Lewkowitz et al., 2019; World Health Organization, 2020b, 2023). Iron‐deficiency in the brain can occur even in the absence of iron‐deficiency elsewhere in the body because the brain is deprioritized relative to the cardiovascular system and other organs (Lewkowitz et al., 2019; World Health Organization, 2020b). Indeed, 37% of pregnant people globally are estimated to be anemic, making this a prevalent and serious issue (World Health Organization, 2023). For all of these reasons, with respect to prenatal nutritional profiles characterized within the DOHaD framework, we focus here on protein‐energy malnutrition and iron deficiency as exemplars within our integrated framework.

## 3 NEURAL SENSITIVE/CRITICAL PERIODS

Neural sensitive/critical periods are learning and adaptation mechanisms foundational to healthy neurodevelopment across species (Greenough et al., 1987; Knudsen, 2004). They reflect carefully‐coordinated processes that create temporal windows of elevated neuroplasticity for learning and adapting to the peri‐ and postnatal environment. That is, neural sensitive/critical periods are “opened” with rapid upregulation of neuroplasticity and “closed” with downregulation of this plasticity by both environmental and neurobiological factors (Takesian & Hensch, 2013; Werker & Hensch, 2015). Notably, sensitive/critical periods are differentiated from each other in part by the degree of neuroplasticity dampening during their closure. Whereas critical periods close with minimal residual plasticity, limiting almost all further learning, sensitive periods close with some remaining neuroplasticity to support continued learning later in development (here we will continue to refer to sensitive/critical periods when both are implicated; Knudsen, 2004; Gabard‐Durnam & McLaughlin, 2020). Sensitive/critical periods significantly impact neurocognitive function in part because the learning that does or does not take place during these windows has persistent effects on neural circuit structure and function and corresponding behaviors over the lifecourse (Greenough et al., 1987; Knudsen, 2004; Takesian & Hensch, 2013). Thus, like the prenatal changes emphasized in DOHaD models, peri‐ and postnatal sensitive/critical periods also robustly impact adult outcomes.

Importantly, neural sensitive/critical periods support learning and adaptation in response to specific properties in the postnatal environment that are species‐ubiquitous (e.g., visual contrast, auditory input; Knudsen, 2004; Greenough et al., 1987). Though, adverse or promotional experiences can alter how and when sensitive/critical periods facilitate learning these ubiquitous environmental cues (Gabard‐Durnam & McLaughlin, 2020; Luby et al., 2020; Nelson & Gabard‐Durnam, 2020). Sensitive/critical periods have been identified to date that serve sensory as well as higher‐order cognitive and affective learning (e.g., language, attachment; Hensch, 2005; Opendak et al., 2017; Werker & Hensch, 2015; Travaglia et al., 2016; Upton & Sullivan, 2010). Moreover, within a domain, multiple sensitive/critical periods can occur to structure and scaffold learning. For example, multiple sensitive periods support language acquisition from infancy through early childhood (see Werker & Hensch, 2015). Given that not all environmental cues and learning domains are subserved by sensitive/critical periods, similarly not every neural circuit or brain region undergoes sensitive/critical periods in postnatal development. Therefore, within our framework and for the remainder of this manuscript, we focus on brain regions known to exhibit sensitive/critical periods, including sensory cortices, the hippocampus, striatum, and the prefrontal cortex (Alberini & Travaglia, 2017; Hensch, 2005; Lieberman et al., 2018; Takesian & Hensch, 2013; Travaglia et al., 2016; Werker & Hensch, 2015; Yang et al., 2012). Importantly, within these brain regions, if the environmental cue to be learned through a sensitive/critical period is not present in the environment when these neuroplasticity windows are open, this learning cannot be fully‐recovered after their closure, leading to lasting differences and deficits (Nelson & Gabard‐Durnam, 2020; Takesian & Hensch, 2013).

Altered opening, closing and overall timing of sensitive/critical periods (even in the presence of appropriate environmental cues) also affects learning with lasting impacts on brain and behavior (Gabard‐Durnam & McLaughlin, 2020; Reh et al., 2020; Takesian & Hensch, 2013). Indeed, much of the empirical work to date has focused on the temporal regulation of these neuroplasticity periods accordingly (e.g., Gabard‐Durnam & McLaughlin, 2020; Reh et al., 2020; Takesian & Hensch, 2013). Neural sensitive/critical period timing is tightly regulated biologically in a number of ways. “Pacers” of these periods serve to prevent precocious maturation, “triggers” initiate this neuroplasticity and learning (i.e., open sensitive/critical periods), and “brakes” dampen plasticity (i.e., close sensitive/critical periods) to preserve what's been learned and structurally shield the neural substrates from future insults (Gabard‐Durnam & McLaughlin, 2020; Hensch & Bilimoria, 2012). Thorough reviews of the vast number of sensitive/critical period regulators may be found elsewhere (e.g., Reh et al., 2020; Takesian & Hensch, 2013). Here, we focus on two key neurochemical and structural regulators of sensitive/critical period plasticity that can be readily examined across species and inform how sensitive/critical periods of learning open and close – neural excitation/inhibition balance and myelination, which we characterize below.

### 3.1 Excitation/inhibition balance as a sensitive/critical period trigger

Developmental changes in brain circuits' balance of neural excitation and inhibition initiate sensitive/critical periods (Hensch & Fagiolini, 2005; Takesian & Hensch, 2013). This excitation/inhibition balance reflects levels of neural communication via the brain's primary excitatory neurotransmitter, glutamate, relative to inhibitory neurotransmitters, including gamma‐Aminobutyric acid (GABA). In particular, normative developmental increase in GABAergic inhibition within a brain circuit serves as a powerful trigger for sensitive/critical period plasticity (Hensch, 2005; Iwai et al., 2003; Toyoizumi et al., 2013). This GABAergic inhibition trigger is so robust that manipulations delivering precocious levels of GABAergic inhibition to a circuit can trigger a sensitive/critical period to start early in the absence of other developmental cues (Takesian et al., 2018; Takesian & Hensch, 2013). Similarly, reduced or delayed GABAergic inhibition levels in a circuit can delay the start of sensitive/critical periods (Hensch, 2005; Takesian & Hensch, 2013). This sensitive/critical period trigger of increasing GABAergic inhibition within circuits is due to the maturation of GABAergic interneurons (inhibitory cells within the brain that secrete GABA; Hensch, 2005).

Importantly for our integrated framework, although these GABAergic interneurons mature postnatally, their number and location within the brain are determined in part prenatally. That is, interneuron developmental precursors are formed and migrate into the cortex *in utero* during late pregnancy in both non‐human and human development (Shenoda, 2017; Xu et al., 2011). There is also a key developmental “functional switch” in how GABA operates within the brain. Namely, before this switch, GABA compounds are depolarizing and thus thought to be largely neuro‐excitatory, whereas after the switch, GABA compounds are hyper‐polarizing and neuro‐inhibitory (and remain so for the duration of the lifecourse; Kilb, 2021; Kirmse & Zhang, 2022; Peerboom & Wierenga, 2021; Zafeiriou et al., 2020). The timing and triggers of this GABA functional switch are under active investigation across species, and this switch may occur perinatally depending on cell‐type, brain region, sex, and species (Peerboom & Wierenga, 2021). However, empirical and clinical evidence (e.g., from GABAergic general anesthetic drug administration in infancy) demonstrate that GABA serves its mature, inhibitory function before the start of postnatal sensitive/critical periods (Reh et al., 2020; Takesian & Hensch, 2013; Toyoizumi et al., 2013). Current findings regarding environmental influences on prenatal GABA function should be interpreted with some caution while the dynamics and timing of this functional switch are clarified. Of course, prenatal environments may also impact GABAergic development independently of the functional switch, for example, by affecting the number of GABAergic neurons, or migration patterns and timing of these cells. In these ways, this key GABA postnatal sensitive/critical period trigger may be significantly influenced by prenatal factors.

The current state of research on excitatory/inhibitory balance in the brain and GABAergic inhibitory development focuses primarily on non‐human animal models. Due to methodological constraints, human work on excitatory/inhibitory balance has relied on post‐mortem studies to date, which are included when available here. Though historically difficult to study in human development, we include this sensitive/critical period regulator because it is such a consistent key feature of sensitive/critical periods and because exciting new methods can now propel postnatal excitatory‐inhibitory balance research forward in the human (Gabard‐Durnam & McLaughlin, 2020). For the scope of this review, we have focused on measures of GABA or glutamate levels/concentration (i.e., including postnatal Magnetic Resonance Spectroscopy or MRS) and counts of GABAergic (e.g., especially parvalbumin‐expressing) or glutamatergic neurons. We have also included studies looking at expression of GABA‐ or glutamate‐related neurotransmitter receptors as these factors also indicate changes in functional GABAergic inhibition or excitation levels in the brain.

### 3.2 Myelination as a sensitive/critical period brake

The second key sensitive/critical period regulator we consider here is myelination, which is a key protective brake that helps end sensitive/critical periods (de Faria et al., 2021). Myelin is a lipid membrane that ensheaths neuron axons to better conduct electrical neural signals and physically protect axons, thus allowing for timely and stable intercellular communication (Bonetto et al., 2021; de Faria et al., 2021; Takesian & Hensch, 2013). Therefore, myelin structurally preserves and protects connections serving learned circuit function during the sensitive/critical period (Hensch & Bilimoria, 2012; McGee et al., 2005; Sivasankaran et al., 2004). Similarly, by physically covering neurons, myelin significantly reduces the kinds and number of potential new connections those neurons can form, effectively significantly reducing plasticity within the circuit and helping to close the sensitive/critical period (Hensch & Bilimoria, 2012; Takesian & Hensch, 2013). Precocious myelination would therefore drive early closure of sensitive/critical periods, while delayed myelination would delay sensitive/critical period closure. Reductions in myelin would similarly delay sensitive/critical period closure and could also result in less‐efficient communication and processing of the learned environmental property, while rendering the neural circuitry vulnerable to future insults and changes (McGee et al., 2005; McGee & Strittmatter, 2003; Takesian & Hensch, 2013). In the central nervous system, myelin is produced by specialized cells called oligodendrocytes (Kuhn et al., 2019). The developmental precursors for oligodendrocytes emerge and migrate prenatally, suggesting they may be influenced by prenatal environmental factors (Bergles & Richardson, 2016; Martínez, 1982; Miller, 2002; Zuchero & Barres, 2013). Though, in both non‐human and human development, oligodendrocytes themselves mature and migrate largely perinatally and postnatally to serve their role as sensitive/critical period “brakes” (Bergles & Richardson, 2016; Martínez, 1982; Miller, 2002; Zuchero & Barres, 2013).

Although myelin can be estimated and quantified in a multitude of ways, for the purpose of this review, we have focused on those most widely used in the literature. Initially discovered using histological techniques (Boullerne, 2016) and then studied in the human using post‐mortem tissue (techniques still in use today), advances in Magnetic Resonance Imaging (MRI) technology have allowed for the quantification of estimates of myelination in vivo in animal models and humans. For histological methods of myelin quantification, we have focused on gene expression of myelin‐related proteins such as myelin basic protein (MBP; Readhead et al., 1990), myelin‐associated glycoprotein (MAG), or proteolipid protein (PLP) as well as the proteins themselves (Bass & Hess, 1969; Inouye & Kirschner, 2016; Norton & Poduslo, 1973). Additionally, because the only known role of oligodendrocytes is in myelin formation for the central nervous system (Kuhn et al., 2019), we took changes in number and development of oligodendrocytes as another marker of myelin. For in vivo measures of myelin, we have focused on the most widely‐used MRI‐based measure, Diffusion Tensor/Weighted Imaging (DTI/DWI), which measures the integrity of white matter microstructure (Heath et al., 2018; Lazari & Lipp, 2021; Mancini et al., 2020).

## 4 INTEGRATING DOHaD AND SENSITIVE/CRITICAL PERIOD FRAMEWORKS

**FIGURE 1:**
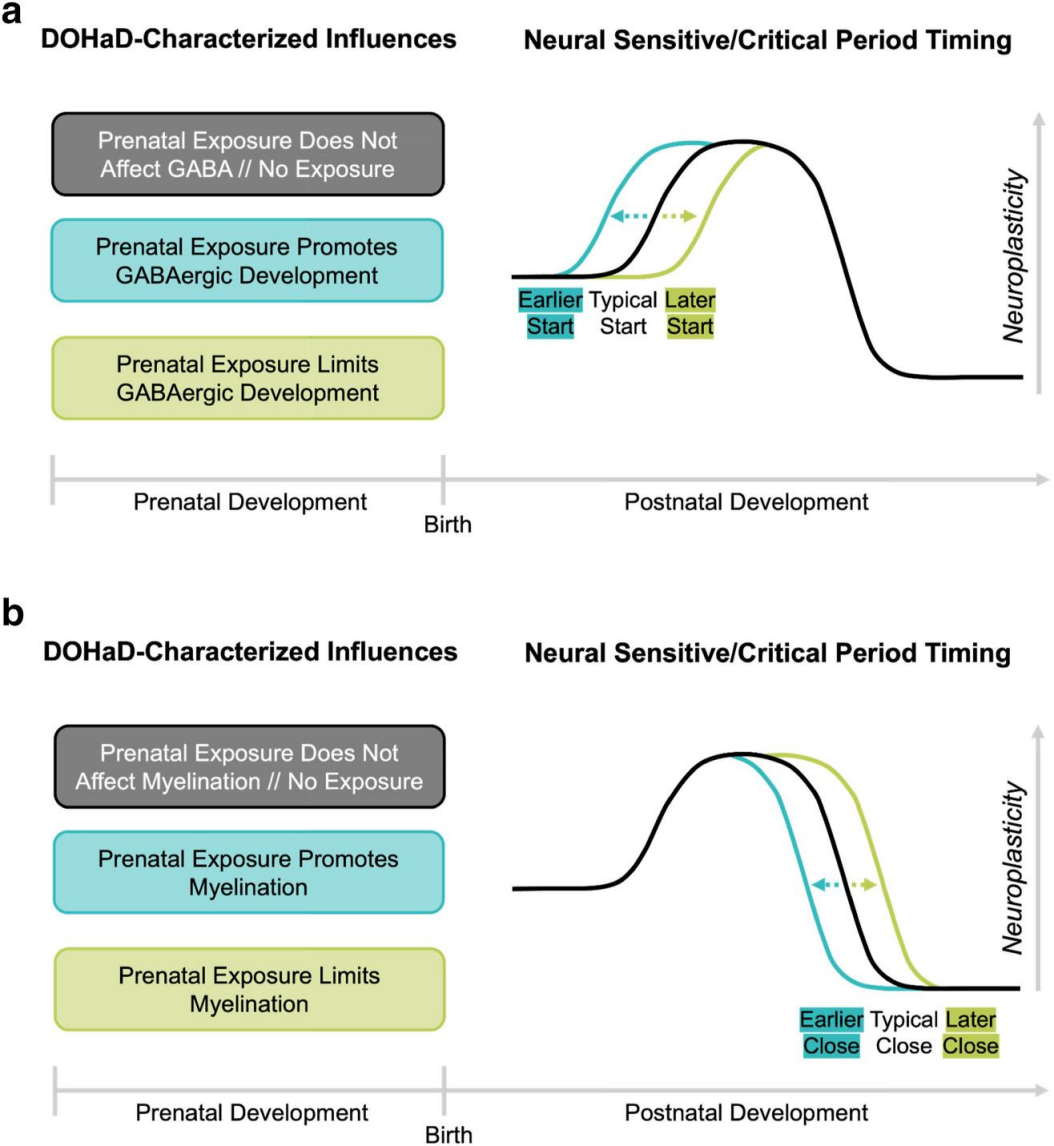
(a) Effects of DOHaD‐characterized prenatal influences on postnatal sensitive/critical period start. Timing of typical sensitive/critical period neuroplasticity indicated in black line. GABAergic development is a sensitive/critical period “trigger” and starts/opens critical/sensitive periods. DOHaD‐characterized prenatal influences with an effect on GABAergic development will alter sensitive/critical period start/opening via corresponding colored lines. That is, prenatal influences that do not affect GABAergic development as well as the lack of a prenatal exposure could likely result in a typical start time (and therefore timing/duration) to the sensitive/critical period. Prenatal exposures promoting GABAergic development could result in an earlier start to the sensitive/critical period, and prenatal exposures limiting GABAergic development could result in a later start to the sensitive/critical period. Coupled with changes in the sensitive/critical period close, these alterations in timing can not only jitter sensitive/critical period timing but also produce changes in the duration of the sensitive/critical period window. (b) Effects of DOHaD‐characterized prenatal influences on postnatal sensitive/critical period close. Timing of typical sensitive/critical period neuroplasticity indicated in black line. Myelination is a sensitive/critical period “brake” and closes critical/sensitive periods. DOHaD‐characterized prenatal influences with an effect on myelination will alter sensitive/critical period close via corresponding colored lines. That is, prenatal influences that do not affect myelination as well as the lack of a prenatal exposure could likely result in a typical close to the sensitive/critical period. Prenatal exposures promoting myelination could result in an earlier close to the sensitive/critical period, and prenatal exposures limiting myelination could result in a later close to the sensitive/critical period. Coupled with changes in the sensitive/critical period start, these alterations in timing can not only jitter sensitive/critical period timing but also produce changes in the duration of the sensitive/critical period window.

We integrate the DOHaD and sensitive/critical period frameworks by examining prenatal environmental influences on postnatal neuroplasticity. That is, prenatal environments induce change and adaptation prenatally but also impact how the brain will change and adapt to postnatal environments through neural sensitive/critical periods of learning. Specifically, we consider how prenatal environments motivated above from the DOHaD framework may impact sensitive/critical period neural substrates to alter when these periods for learning occur postnatally (integrated model illustrated in Figure 1). To demonstrate the potential utility of this integrated framework, we provide empirical evidence for how sensitive/critical period substrates of excitatory/inhibitory balance (Figure 1a) and myelination (Figure 1b) are impacted by DOHaD‐characterized prenatal experiences within the following categories as illustrative examples: (1) substance exposures, both psychoactive substances including alcohol, nicotine, cocaine, or cannabis and environmental pollutants including lead, cadmium, mercury, and arsenic; and (2) prenatal nutrition, both protein‐energy malnutrition and iron deficiency. To provide the most complete picture of these prenatal effects across levels of analysis, we include evidence from both non‐human animal model and human neurodevelopment whenever available.

**FIGURE 2:**
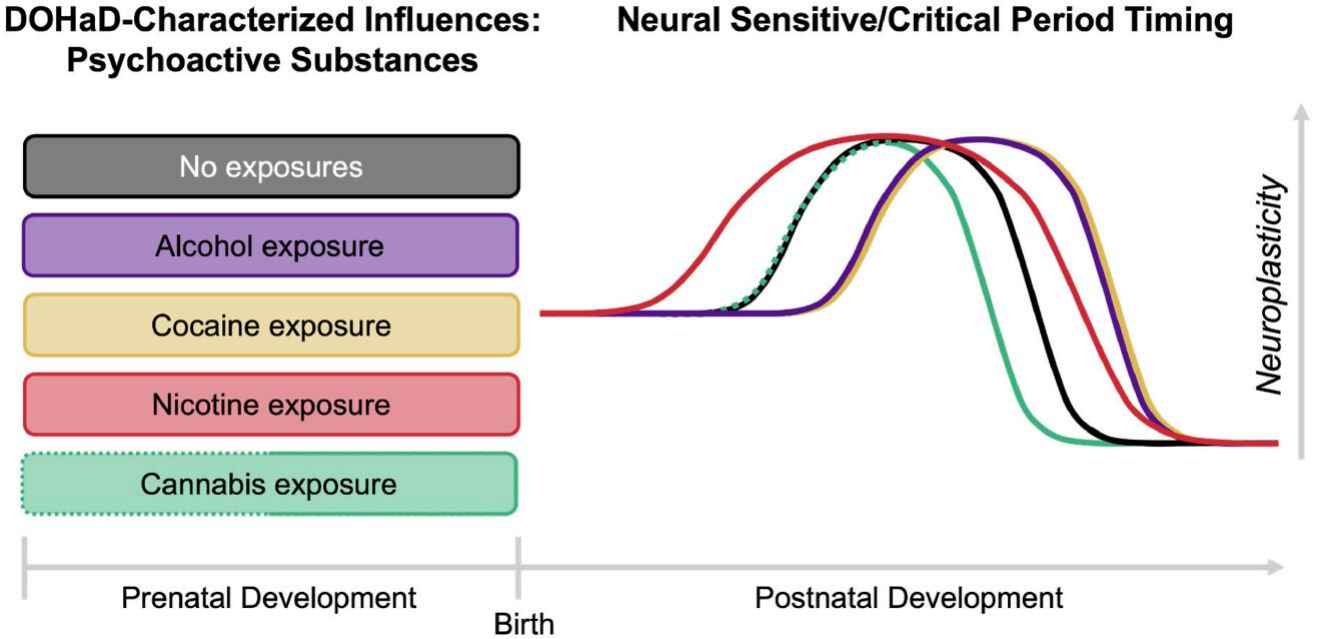
Effects of prenatal psychoactive substance exposures on projected postnatal sensitive/critical periods timing. Timing of typical sensitive/critical period neuroplasticity indicated in black line (the no exposures condition). Extrapolated effects from extant literature of each DOHaD‐characterized prenatal psychoactive substance on sensitive/critical period timing and duration shown in corresponding colored lines. Dotted lines indicate preliminary evidence for effect that requires further research and verification. For example, prenatal alcohol exposure may result in the limitation of GABAergic development and myelination, thus leading to a delay in the start and closure of sensitive/critical periods. With prenatal alcohol exposure, the uniform delay of both the start and end of the sensitive/critical period would likely preserve the typical duration of the sensitive/critical period window.

**FIGURE 3:**
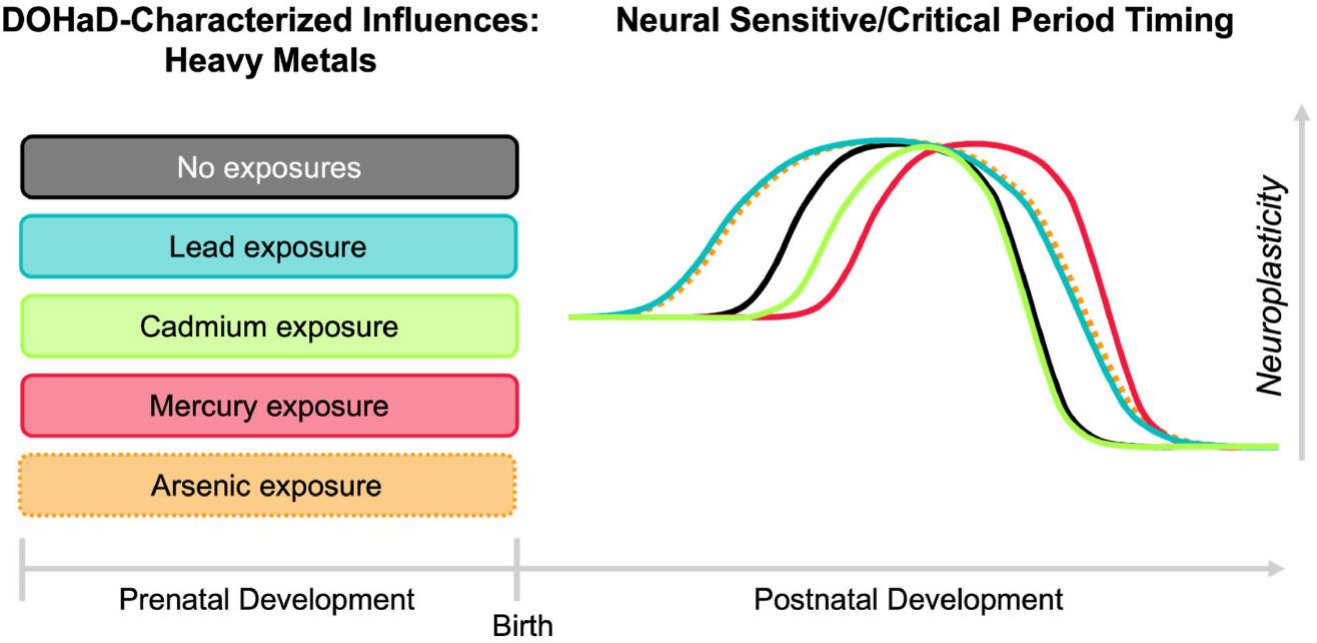
Effects of prenatal heavy metal exposures on projected postnatal sensitive/critical periods timing. Timing of typical sensitive/critical period neuroplasticity indicated in black line (the no exposures condition). Extrapolated effects from extant literature of each DOHaD‐characterized prenatal heavy metal exposure on sensitive/critical period timing and duration shown in corresponding colored lines. Dotted lines indicate preliminary evidence for effect that requires further research and verification. For example, prenatal lead exposure may result in the promotion of GABAergic development and the limitation of myelination, thus leading to an earlier start of the sensitive/critical period window and delayed closure. With prenatal lead exposure, this earlier start and later end would likely increase the duration of the sensitive/critical period window.

**FIGURE 4:**
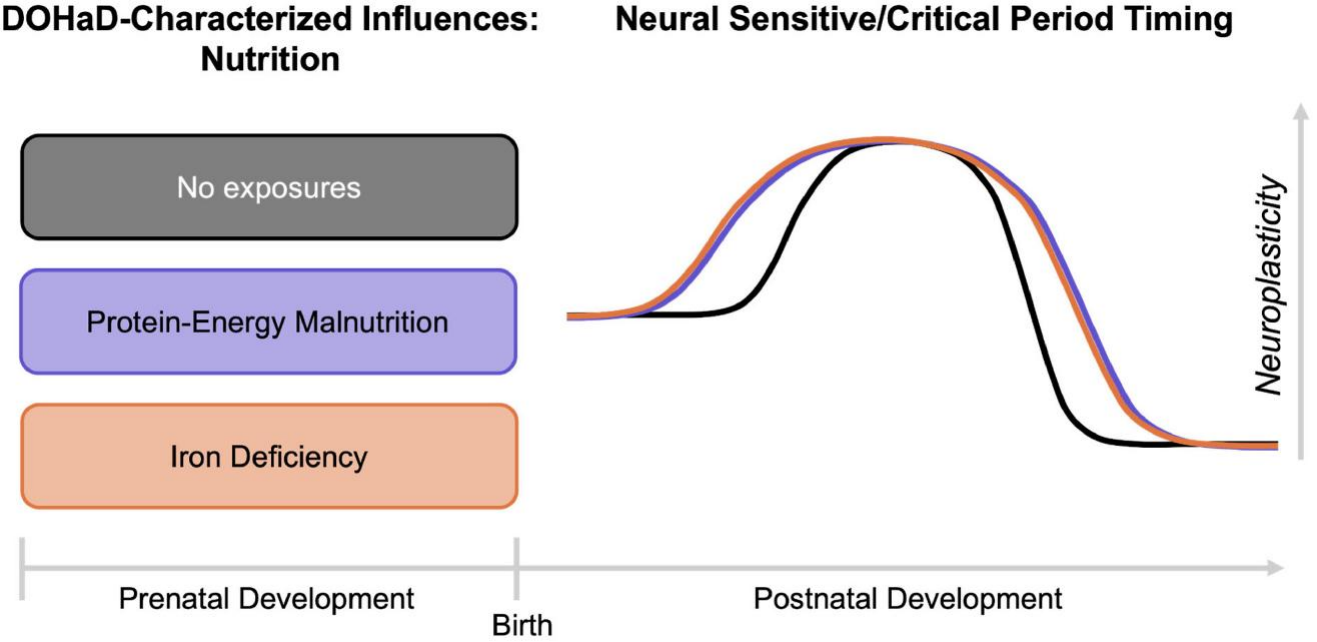
Effects of prenatal nutrition on projected postnatal sensitive/critical periods timing. Timing of typical sensitive/critical period neuroplasticity indicated in black line (the no exposures condition). Extrapolated effects from extant literature of each DOHaD‐characterized prenatal nutritional profile on sensitive/critical period timing and duration shown in corresponding colored lines. Dotted lines indicate preliminary evidence for effect that requires further research and verification. For example, in utero exposure to iron deficiency may result in the promotion of GABAergic development and the limitation of myelination, thus leading to an earlier start of the sensitive/critical period window and delayed closure. With the exposure of prenatal iron deficiency, this earlier start and later end would likely increase the duration of the sensitive/critical period window.

We also note that multiple categories of prenatal influences can co‐occur with additive, interactive, independent, or synergistic effects that will not be considered within the scope of the present evidence (e.g., Goldstein et al., 2017; Nakhid et al., 2022; Reid et al., 2021; Reid & Georgieff, 2023). For example, a fetus with exposure to prenatal alcohol may also be co‐exposed to insufficient iron *in utero* (Nakhid et al., 2022). At the same time, we assert that co‐occurrence of prenatal exposures does not preclude understanding differential effects of specific experiences on subsequent neurodevelopment within our framework, and here we focus on these differential effects (Dufford et al., 2021; McLaughlin et al., 2021).

Finally, empirical studies directly testing how prenatal experiences alter postnatal sensitive/critical period timing and learning are currently extremely rare (e.g., Weikum et al., 2012), a motivating factor in creating this conceptual framework. However, given the evidence for how each prenatal environmental factor influences the sensitive/critical period substrates of excitation/inhibition balance and myelination, we provide posited changes in sensitive/critical period timing to guide new empirical studies moving forward. If insufficient or inconsistent evidence exists to make such a projection for any prenatal factor, this is also noted in text and in illustrative figures.

### 4.1 Prenatal substance exposures

Looking beyond the teratogenic effects (Minnes et al., 2011) of certain prenatal substance exposures affecting morphological critical periods occuring in utero, exposure to substances also affect neurobiological regulators of sensitive/critical period timing postnatally in the following ways.

#### 4.1.1 Psychoactive substances: Alcohol, nicotine, cocaine, and cannabis

##### Impacts on excitatory/inhibitory balance

There is strong evidence that exposure to alcohol prenatally disrupts the GABAergic system (Isayama et al., 2009; Shenoda, 2017). In late gestation mice models of prenatal alcohol exposure (i.e., alcohol exposure on postnatal day 7 which is equivalent to late gestation in the human), researchers found reduced GABAergic (including parvalbumin‐positive) neurons across the adult brain (Smiley et al., 2015), including both reduced GABAergic neuron and excitatory pyramidal neuron counts in the adult frontal cortex (though GABAergic neurons were reduced more severely; Coleman et al., 2012). Other studies of rodents with exclusively *in utero* alcohol exposure found reduced migration of GABAergic cells into the cortex during the embryonic period (Cuzon et al., 2008) with consequences for the adult brain (e.g., hippocampus; Madden et al., 2020). A human post‐mortem study examining brain tissue from 15 weeks gestation to 22 postnatal months in those with or without prenatal alcohol exposure with immunohistochemical approaches has corroborated these findings (Marguet et al., 2020). Specifically, they observed delayed and insufficient production of GABAergic neurons in the tissue of those with prenatal alcohol exposure relative to those without exposure, leading to inadequate numbers and mispositioning of GABAergic neurons in the postnatal human prefrontal cortex (Marguet et al., 2020). This evidence across species consistently indicates that prenatal alcohol exposure delays GABAergic neuron development and reduces GABAergic neurons postnatally, both of which would in turn delay sensitive/critical period timing postnatally.

Similarly, studies have identified disruptions to GABAergic development following *in utero* cocaine exposures. Specifically, prenatal cocaine exposure leads to reduced numbers of GABAergic neurons in the cerebral cortex of embryonic rodent brains, likely due to observed reductions in inhibitory cell migration prenatally (Crandall et al., 2004; McCarthy et al., 2011). There is some evidence prenatal cocaine may also affect excitatory cells *in utero* (Lee et al., 2011) though disruption in the migration of GABAergic neurons and decreased cortical GABAergic neuron numbers in the same study suggest that GABAergic function is more significantly impacted. Similar disruptions to the GABAergic system were observed in the medial prefrontal cortex of young adult mice with prenatal cocaine exposure, including decreased GABAergic neuron numbers and GABA‐to‐projection neuron ratio (McCarthy & Bhide, 2012). The similar pattern of results across the lifespan suggest lasting GABAergic deficits as a result of prenatal cocaine exposure. Thus, prenatal cocaine exposure seems to reduce GABAergic cell number postnatally, which would delay sensitive/critical period timing and learning subserved by these mechanisms.

In contrast to these other substances, most work examining the effects of prenatal nicotine exposure on neural excitation/inhibition balance suggest that this exposure leaves GABAergic inhibition development intact (although see Martin et al., 2020). Instead, excitatory (i.e., glutamatergic) function seems impacted by prenatal nicotine exposure. For example, rats prenatally exposed to nicotine exhibit decreased levels of glutamate‐related proteins in the juvenile period (e.g., AMPA receptor subunit GluR1) in the hippocampus (Parameshwaran et al., 2012). Similar effects were observed in the prefrontal cortex of prenatally exposed peripubertal mice, with reduced gene expression of glutamate‐related genes, although the specific gene affected was dependent on sex (Polli et al., 2020). These effects persist through adulthood; adult mice prenatally exposed to nicotine have also demonstrated decreased glutamatergic neuron density in the prefrontal cortex (Aoyama et al., 2016). Thus, prenatal nicotine exposure shifts excitatory/inhibitory balance towards greater relative inhibition, which may precociously start sensitive/critical periods. Reduced excitatory communication due to prenatal nicotine exposure likely also impairs learning during sensitive/critical periods.

Finally, research targeting prenatal exposure to cannabis indicates effects on excitatory/inhibitory balance, though the direction of the net effect remains unclear. Current research on this topic primarily focuses on the hippocampus (Bara et al., 2021; Higuera‐Matas et al., 2015; Hurd et al., 2019). Pre‐ and perinatal cannabis exposure has been shown to impair GABAergic neuronal function in the hippocampus for infant/juvenile rodents (Vargish et al., 2017) that persists into adulthood (Beggiato et al., 2017). Moreover, in a late gestation rat model of cannabis exposure (i.e., exposure in early rodent postnatal period, equivalent to late human gestation) researchers found a delay in the developmental “switch” for GABA when it begins to exert inhibitory effects in the prefrontal cortex in exposed rats compared to non‐exposed rats (Scheyer et al., *in press*). However, rats prenatally exposed to cannabis have also demonstrated decreased hippocampal glutamate levels as juveniles and adults (Mereu et al., 2003). Together this literature provides clear evidence that excitatory/inhibitory balance is impacted by prenatal cannabis exposure. However, how prenatal cannabis exposure changes this balance and the net impact on sensitive/critical period timing remains to be clarified.

##### Impacts on myelination

Prenatal exposure to these drugs/substances may also impact myelination in postnatal development responsible for closing sensitive/critical periods. Evidence across studies of prenatal alcohol and nicotine exposures shows these substances impact the sensitive/critical period brake myelin by impairing myelination (i.e., dysmyelination). Specifically, there exists a rich literature characterizing the dysmyelinating effects of prenatal exposure to alcohol in vitro (Darbinian & Selzer, 2021), in animal (Almeida et al., 2020; Darbinian & Selzer, 2021), and in human (Donald et al., 2015; Wozniak & Muetzel, 2011) research. Similarly, research using rodent and zebrafish models indicate that prenatal exposure to nicotine results in altered myelin‐related gene expression, including impaired myelin especially in females within the prefrontal cortex (Cao et al., 2013; Omotoso et al., 2015; Zhao et al., 2013). In human development, MRI‐based diffusion tensor imaging corroborates impairments in white matter integrity for human infants and adolescents with prenatal nicotine exposure, suggesting persistent impacts on myelination (Chang et al., 2016; Liu et al., 2011; Paus et al., 2008). However, two of these human developmental studies (Chang et al., 2016; Liu et al., 2011) looked at *in utero* nicotine exposure in conjunction with another substance, highlighting a persistent limitation of conducting research on prenatal substance exposure with humans (Lebel & Sowell, 2011; Margolis et al., *in press*). Thus, current evidence indicates both prenatal alcohol and nicotine exposures may interfere with sensitive/critical period closure by delaying or preventing typical end to plasticity and may not robustly protect the physical connections made during sensitive/critical period learning.

Initial evidence suggests prenatal cocaine exposure also impairs myelin development, though additional research is needed across species (Gkioka et al., 2016). As far as we are aware, there are only two studies directly measuring prenatal cocaine exposure's impact on myelin in animal models. Specifically, rat pups perinatally exposed to cocaine (i.e., during gestation and lactation) have exhibited reduced concentrations of myelin compared to controls (Wiggins & Ruiz, 1990). Similarly, rat pups prenatally exposed to cocaine and subjected to MRI‐based diffusion tensor imaging have demonstrated decreased fractional anisotropy values, though paired only with significant decreases in axial diffusivity, this evidence might suggest axonal damage rather than impairments in myelin itself (McMurray et al., 2015). Other animal studies suggest impairments to myelination using auditory brainstem responses, though this evidence is indirect without specific myelin measurements (Gkioka et al., 2016). A human study of prenatal cocaine exposure also using MRI‐based diffusion tensor imaging found increases in axial diffusivity in frontal white matter pathways in children, consistent with impaired myelination (Warner et al., 2006; though see null effects in other frontal pathways: Liu et al., 2011). Participants in both of these human studies also had other concurrent prenatal drug exposures that may explain their discrepant results. Though further research is needed, initial evidence points to prenatal cocaine exposure impairing myelination and delaying sensitive/critical period closure.

There is a dearth of knowledge across species on the effects of prenatal cannabis exposure with respect to myelin development. We found no studies to date in non‐human animals or humans addressing this question. However, evidence from rodent research on cannabis exposure during the early postnatal period (Arévalo‐Martín et al., 2007; Huerga‐Gómez et al., 2021) and in rodent models of disease treated with cannabinoids (Arévalo‐Martín et al., 2003; Solbrig et al., 2010) suggests that prenatal cannabis exposure may promote myelination. Downstream, this class of exposure may result in early closure of sensitive/critical period windows, but research is urgently needed targeting prenatal cannabis exposure.

##### Psychoactive substances summary

In summary, prenatal exposures to psychoactive substances have significant and differing effects on the sensitive/critical period substrates that regulate timing of these windows for learning postnatally (see Figure 2). Integrating across robust effects observed on both sensitive/critical period excitatory/inhibitory balance triggers and myelination brakes, prenatal alcohol exposure would putatively delay both the start and closure of sensitive/critical periods, indicating a general delay in early learning. Prenatal cocaine exposure appears to have the same effects as alcohol, and may also delay sensitive/critical period timing, though further evidence for how prenatal cocaine impacts neural regulators of sensitive/critical period closure is needed. In contrast, prenatal nicotine exposure may cause sensitive/critical periods to begin too early while closing too late, which would lead to both altered timing and altered duration of sensitive/critical period learning. Much further research is needed examining prenatal cannabis exposure as the net impact on sensitive/critical period initiation is currently unresolved, while initial findings suggest cannabis may close sensitive/critical periods early.

#### 4.1.2 Environmental pollutants: Heavy metals (lead, cadmium, mercury, and arsenic)

##### Impacts on excitatory/inhibitory balance

Though the literature on *in utero* heavy metal exposures is less rich than for psychoactive substances, there is consistent evidence that heavy metal exposures do differentially impact postnatal excitatory/inhibitory balance regulating sensitive/critical period initiation. For example, two studies have used parallel methods to examine the effect of lead or cadmium exposure during gestation and lactation on GABA and glutamate concentrations in rat pup brains (Antonio et al., 2010; Leret et al., 2002). In this pair of studies, lead and cadmium perinatal exposures appear to alter postnatal excitatory/inhibitory balance in the cerebral cortex in opposite ways (Antonio et al., 2010; Leret et al., 2002). Specifically, perinatal lead exposure led to a decrease in glutamate concentration in both the cerebral cortex and hippocampus and a smaller decrease in GABA concentrations within the cortex only, suggesting overall effects of increased relative inhibition in these regions (Leret et al., 2002). Perinatal cadmium exposure was linked to increased glutamate concentrations in the hippocampus and reduced GABA levels in prefrontal cortex, consistent with overall effects of greater excitatory relative to inhibitory balance across cortical areas (Antonio et al., 2010). Similarly, one study has found that methylmercury exposure during gestation and lactation leads to a reduction in GABA levels and elevation in glutamate levels in male rat pups (Ben Bacha et al., 2020). With respect to arsenic exposure, there is an urgent need for literature examining exclusively prenatal or maternal transmission (i.e., via lactation). Although, one study examining arsenic exposure beginning prenatally and continuing until post‐weaning has observed increased gene expression of GABAergic interneuron markers (i.e., glutamate decarboxylase (GAD)65 and GAD 67) in juveniles' cortices and hippocampi as compared to non‐exposed pups, suggesting increased relative inhibition in these regions (Xi et al., 2010). Thus, initial evidence for each heavy metal indicates early exposure can impact postnatal excitatory/inhibitory balance regulators of sensitive/critical periods.

##### Impacts on myelination

*In utero* heavy metal exposures also impact postnatal myelination brakes on sensitive/critical period plasticity. Of the four heavy metals considered here, prenatal exposure to lead, mercury, and arsenic all have initial evidence for disrupting myelin formation (Naffaa et al., 2021). Cadmium is implicated in deleterious effects on myelin, but primarily has a demyelinating effect (i.e., harming extant myelin that has already developed) rather than a dysmyelinating one (i.e., impairing myelin development; Naffaa et al., 2021). Human studies examining how lead exposure disrupts myelin development are limited to postnatal rather than prenatal lead exposure but demonstrate impaired myelin lasting into adulthood (Brubaker et al., 2009; Cecil, 2011; though see Migneron‐Foisy et al., 2022). Moreover, one study of low‐level prenatal lead exposure in humans provides indirect evidence for altered myelin development, finding delayed auditory evoked brain responses consistent with impaired or delayed myelination in infant sensory cortices (Silver et al., 2016). Consistent with these human studies, considerable work with animal models suggests in utero exposure to lead disrupts myelination processes (Deng & Poretz, 2001; Latronico et al., 2022; Nam et al., 2019a, 2020). Prenatal mercury exposure in animal models downregulates expression of myelin basic protein, suggesting impaired myelin development as well (da Silva et al., 2022; Heimfarth et al., 2018). A single study in human adolescents has also found deleterious effects of prenatal mercury exposure on MRI‐based diffusion tensor imaging measures of the corpus callosum in frontal cortex (Migneron‐Foisy et al., 2022). Limited studies examine arsenic exposure constrained to the prenatal period, even in animal models, although arsenic exposure beginning *in utero* and continued postnatally was related to impaired myelin development (Niño et al., 2020; Ríos et al., 2009; Zarazúa et al., 2010). Thus studies of prenatal lead, mercury, and arsenic exposures all point to impaired myelin development and potentially delayed sensitive/critical period closures.

##### Environmental pollutants summary

Early evidence indicates prenatal exposures to environmental pollutants like heavy metals impacts biological regulators governing both the start and close of sensitive/critical periods postnatally (see Figure 3). Integrating across the excitatory/inhibitory balance and myelination effects, prenatal lead exposure may begin sensitive/critical periods early and delay their closure, disrupting their timing and duration. Prenatal cadmium exposure may delay the start of sensitive/critical periods and without any documented impact on their closure, this exposure may ultimately shorten the duration of postnatal sensitive/critical periods. Prenatal mercury exposure may delay both the start and closure of sensitive/critical periods, shifting them in time but preserving their duration. Evidence for impacts of prenatal arsenic exposure is still very sparse, but tentatively this exposure may start sensitive/critical periods early and delay their closure, disrupting both timing and duration (similarly to lead). Further research across species is needed to shore up this initial evidence across heavy metals, however.

### 4.2 Prenatal nutrition

The second class of DOHaD‐characterized prenatal environmental factors we assess in the context of our integrated framework are prenatal nutritional profiles. Specifically, we review the state of evidence for how prenatal protein‐energy malnutrition and iron deficiency each shape excitatory/inhibitory balance and myelination regulators of sensitive/critical periods postnatally.

#### 4.2.1 Protein‐energy malnutrition

##### Impacts on excitatory/inhibitory balance

Protein‐energy malnutrition during gestation has been implicated in alterations in the GABAergic system, with much research focusing on the hippocampus (Morgane et al., 2002). In rodent models of prenatal protein‐energy malnutrition, this exposure increases GABAergic inhibition within the hippocampus (Bronzino et al., 1999), and adult rats show altered GABAergic regulation in this region (Steiger et al., 2003). Differential effects of prenatal protein malnutrition dependent on hippocampal subregion were also observed when examining the number of GABAergic interneurons over development. In two separate studies, adolescent rats with prenatal protein malnutrition had an increased number of GABAergic interneurons in the dentate gyrus area of the hippocampus compared to controls, consistent with increased inhibitory function (Díaz‐Cintra et al., 2007; González‐Maciel et al., 2015). To our knowledge, no human developmental studies have yet examined whether protein‐energy malnutrition prenatally has similar effects on postnatal excitatory/inhibitory balance changes to favor increased inhibitory function, however.

##### Impacts on myelination

Surprisingly, the literature examining the effect of protein‐energy malnutrition exclusively in the prenatal (or perinatal pre‐weaning) period on myelination is somewhat limited across species. Mice with *in utero* undernutrition and then cross‐fostered to receive adequate nutrition after birth exhibited decreased myelin yield from a juvenile age through adulthood (Berkow & Campagnoni, 1983) as well as decreased levels of important myelin components (e.g., galactolipids; Berkow & Campagnoni, 1981). Rats reared with the same paradigm of undernutrition followed by cross‐fostering have similarly demonstrated thinner myelinated fiber diameters as well as a higher density of unmyelinated fibers as compared to controls (Olivares et al., 2012). Studies with human cohorts conceived during famines such as the Dutch Famine of 1944–1945 provide an opportunity to follow the effects of potential protein‐energy malnutrition in humans in vivo (De Rooij et al., 2022). However, one study found that exposure to the Dutch famine was unrelated to MRI‐based DTI measures of mean diffusion and fractional anisotropy in older adulthood (De Rooij et al., 2016). Whether prenatal malnourishment‐related differences occurred during postnatal development remains untested in the human, however.

#### 4.2.2 Iron deficiency

##### Impacts on excitatory/inhibitory balance

Prenatal iron deficiency is linked to both changes in excitation and inhibition postnatally (Kim & Wessling‐Resnick, 2014). The hippocampus and cortex are both highly sensitive to changes in prenatal iron levels (Beard & Connor, 2003). Specifically, rodent research has found decreased excitation as well as accelerated GABAergic development, suggesting increased relative inhibition levels and decreased overall plasticity for learning (Kim & Wessling‐Resnick, 2014). Rats with perinatal iron deficiency and later iron supplementation also exhibited altered glutamate and glutamine levels associated with reduced excitatory communication (measured with MRI‐based spectroscopy or MRS) in the striatum at two time points as pups, but not in the peripubertal stage (Ward et al., 2007). Perinatal iron deficiency has also led to immaturities in rat hippocampal pyramidal cell morphology (Jorgenson et al., 2003). Genetic manipulations in the rodent hippocampus have further specified that excitatory neuron deficits and excitatory communication deficits are due to lack of iron rather than general anemic effects (Carlson et al., 2009). With respect to GABAergic inhibition, male rats with perinatal iron deficiency have demonstrated precocious GABAergic maturation compared to controls across development (Callahan et al., 2013). Although this effect was not replicated in (Boksa et al., 2017), this may be due to this team's earlier assessment time points which overlapped with windows of null effects in the prior study. One study has evaluated both excitatory and inhibitory alterations using MRI‐based spectroscopy (i.e., MRS) in rats with perinatal iron deficiency (i.e., gestation through postnatal day 7, equivalent to entire human gestation period), finding altered levels of glutamate and GABA in the hippocampus across development that together suggest reduced glutamatergic functioning and increased GABAergic inhibition, respectively (Rao et al., 2003). Taken together, prenatal iron deficiency impacts both excitatory and inhibitory communication to shift this balance towards inhibition, which may start sensitive/critical periods early in postnatal development.

##### Impacts on myelination

Iron serves a critical role in myelination as a co‐factor for synthesizing key myelin components (Connor & Menzies, 1996). The consequences of prenatal iron deficiency on myelination are therefore well‐documented in animal models across multiple levels of analysis (Lozoff & Georgieff, 2006). Rat pups with perinatal iron deficiency have shown downregulation of six myelin‐related genes (i.e., myelin‐associated oligodendrocytic basic protein, myelin and lymphocyte protein, myelin oligodendrocyte glycoprotein, myelin basic protein, proteolipid protein, peripheral myelin protein 22) in postnatal development (Clardy et al., 2006). In adulthood, decreased myelin content and myelin‐related proteins have been observed following prenatal iron deficiency (Ortiz et al., 2004). A few studies have suggested regional specificity to the impacts of prenatal iron deficiency (e.g., Morath & Mayer‐Pröschel, 2002; Wu et al., 2008). However, to our knowledge, the only animal studies using MRI techniques to examine white matter microstructural integrity examine the effect of early life iron deficiency rather than during gestation or gestation/lactation (e.g., see MRI‐based diffusion tensor imaging of pigs in Mudd et al., 2018, and of non‐human primates in Vlasova et al., 2021). To date, the only human study measuring myelin in relation to prenatal iron status (as measured by self‐report for the third trimester and cord blood ferritin) found that neonates with decreased gestational iron had decreased white matter microstructural integrity using MRI‐based diffusion tensor imaging (Monk et al., 2016). Indirect evidence in human development following prenatal iron deficiency using auditory‐evoked brain responses is also consistent with delayed or impaired myelination postnatally (Amin et al., 2013). Thus, there is consistent evidence across species that prenatal iron deficiency impairs postnatal myelination, which would delay sensitive/critical period closure and may not robustly protect the physical connections made during sensitive/critical period learning thereafter.

#### 4.2.3 Prenatal nutrition summary

Evidence from both protein energy malnutrition and iron deficiency as prenatal nutrition factors indicate consistent effects on postnatal sensitive/critical period biological regulators (see Figure 4). That is, both prenatal protein energy malnutrition and iron deficiency are projected to start sensitive/critical periods earlier in development and close them later with a delay due to impaired myelination.

### 4.3 Limitations of existing evidence

The extant evidence presented above suggests many of the prenatal factors characterized by the DOHaD framework have significant impacts on sensitive/critical period substrates and therefore may influence sensitive/critical period timing and learning postnatally as well. Although this body of evidence indicates the integration of DOHaD and sensitive/critical period frameworks in this way would provide novel insight into early biological embedding phenomena, there are several limitations in the extant evidence worth noting below that should be addressed in future empirical studies.

First, though there is substantial evidence that prenatal exposures impact postnatal sensitive/critical period regulators, very little research to date has carefully characterized the conditions under which those prenatal exposures exert their influence. We therefore urge researchers studying exposures in the prenatal period across species to carefully characterize the nature of exposures in several ways. First, care should be taken to manipulate in non‐human models and measure in humans the dose, timing (e.g., trimester of exposure), and pattern (e.g., presence of binge‐drinking behaviors for alcohol) of exposures in the prenatal period (Grandjean et al., 2008; Margolis et al., *in press*; while also balancing participant burden in human research). For example, measurement in human studies can range from very detailed interviews such as the Timeline Followback Interview (Martin‐Willett et al., 2020; Sobell et al., 1979) to multiple choice questionnaires or single yes/no elections. Moreover, non‐human animal model research should titrate, or at least note in manuscripts, whether an exposure dose tested could potentially map to levels experienced in human development whenever possible, though we acknowledge how challenging dose titration can be between species, especially when human exposure is poorly characterized for a particular substance. Prenatal alcohol exposure is an exception where extensive effort has been made to characterize these exposure dimensions, with research documenting neurodevelopmental differences related to dosage (e.g., Almeida et al., 2020; Flak et al., 2014; Jacobson et al., 2017), timing (e.g., Scher et al., 1998), and pattern (e.g., Flak et al., 2014; Kelly et al., 1987; West et al., 1987). Other substances and nutritional factors should undergo similarly rigorous accounting in human studies and experimental manipulation in animal models.

A second consideration in characterizing exposures is that often research participants experience multiple exposures during the prenatal period. Here, we cannot overstate the utility of animal models to provide greater experimental control to identify interactive, synergistic, or additive impacts of complex exposure profiles. In addition, exciting work from in vitro and organoid models can provide evidence working to disentangle multiple clusters of exposures on specific brain components such as myelin (Chesnut et al., 2021a, 2021b). Though some animal and organoid model research has begun testing such effects, much more research is needed in this space to build translational knowledge that informs the real‐world complex prenatal exposures most frequently found in humans. In much of the current human literature, sample sizes are too small to effectively disentangle effects of each exposure, confounding interpretations of differential, specific effects. However, large‐scale human studies such as the HEALthy Brain and Child Development Study (HBCD) in the United States will soon provide researchers with the sample size to investigate both differential and synergistic effects of multiple prenatal substance exposures (Jordan et al., 2020; Morris et al., 2020; Volkow et al., 2021). We can also look to other fields for innovative statistical techniques to disentangle multiple exposures such as the mixture model from the field of environmental toxicology which is used to examine outcomes associated with multiple co‐occurring environmental chemical exposures (overview of method: Liu, Bobb, Claus Henn, Gennings, et al., 2018; Liu, Bobb, Claus Henn, Schnaas, et al., 2018; applied to prenatal alcohol exposure: Pini et al., 2019). Thus, moving forward, we hope to see more careful characterization of prenatal exposures, both as singular exposures and as complex exposure combinations, when testing how these exposures become biologically embedded in neurodevelopment.

With respect to the neural measurements within this empirical body of work, we note that while studies in human development were present for the vast majority of the prenatal factors considered, human evidence for changes to excitatory/inhibitory balance were relatively sparse compared to evidence concerning myelination. Historically, in vivo measurement of human excitatory/inhibitory balance changes over development was almost entirely infeasible, contributing to this paucity of evidence. However, recent technological and computational advances have provided ways to address these limitations. Specifically, MRI‐based magnetic resonance spectroscopy (MRS) now provides ways to extract in vivo concentrations of both excitatory (glutamate) and inhibitory (GABA) neurotransmitters within particular brain regions from infancy through adulthood (Buonocore & Maddock, 2015; Kwon et al., 2014; Perdue et al., 2023; Puts & Edden, 2012; Van der Graaf, 2010). MRS of course has its own limitations, for example, GABA estimation can have low signal‐noise properties with some extraction measures (Puts & Edden, 2012) and relatively large voxel sizes are still required such that spatial specificity is somewhat limited. Still, the MRS approach provides an exciting opportunity to image in vivo neurochemical changes over human development to address the limited current evidence for how prenatal factors influence this sensitive/critical period substrate postnatally.

Finally, it is important to note that while excitatory/inhibitory balance and myelination have particularly well‐characterized roles in sensitive/critical period regulation, they are just two of many factors that govern these periods' timing and learning (Reh et al., 2020; Takesian & Hensch, 2013). Thus there are bound to be additional effects of the prenatal environmental factors considered here on sensitive/critical period mechanisms that were not described in this empirical review. Such additional effects may exacerbate or counter the projected impacts on sensitive/critical period opening and closing due to changes in excitatory/inhibitory balance and myelination that we provided in this review. While we included these projected changes to sensitive/critical period timing to provide specific, testable target hypotheses for future research, undoubtedly some of these projections will thus prove false or more complex changes to sensitive/critical periods will be uncovered with additional empirical study.

## 5 FUTURE RESEARCH DIRECTIONS FOR IMPLEMENTING AN INTEGRATED FRAMEWORK OF BIOLOGICAL EMBEDDING

Future research should continue to expand the body of evidence presented above with respect to how prenatal environmental factors impact key sensitive/critical period substrates. However, our integrated framework of biological embedding also reveals several new directions to drive research moving forward across species. Below we highlight two such opportunities.

### 5.1 Testing prenatal environments' impacts on sensitive/critical period learning

We hope to spur research moving forward that directly tests how these prenatal experiences impact sensitive period opening, learning, closing, and the behavioral consequences of these changes across species. The empirical evidence presented here provides robust foundational understanding for how prenatal experiences can impact postnatal sensitive/critical period substrates in brain regions known to have sensitive/critical periods (e.g., sensory cortices like visual and auditory cortex, the hippocampus, striatum, and the prefrontal cortex; Alberini & Travaglia, 2017; Hensch, 2005; Lieberman et al., 2018; Takesian & Hensch, 2013; Travaglia et al., 2016; Werker & Hensch, 2015; Yang et al., 2012). Though this body of work largely stops short of testing how these changes to neural substrates impact the sensitive/critical period itself in terms of timing and learning, the extant evidence motivates studies exploring specific sensitive/critical period changes and behavioral sequelae moving forward. We and others have elsewhere reviewed approaches to test sensitive/critical periods in multiple species to guide such studies (e.g., Gabard‐Durnam & McLaughlin, 2020; Reh et al., 2020; Takesian & Hensch, 2013; Werker & Hensch, 2015). Importantly, while there is currently limited longitudinal evidence from human studies, robust testing of critical/sensitive period changes will require longitudinal designs moving forward to substantiate claims about altered timing and duration of these periods. Preliminary longitudinal research in both rodent models (e.g., St. Pierre et al., 2021) and humans (e.g., Weikem et., 2012) that do directly test prenatal environmental influences on postnatal sensitive/critical period timing and learning support our framework's feasibility and potential to reveal new insights into early neurodevelopment.

### 5.2 Identifying mitigating and buffering prenatal factors on postnatal neuroplasticity

We also hope to see research begin to target potential exposures prenatally or postnatally that can mitigate or buffer against some of the documented effects of substance and nutritional factors on sensitive/critical period substrates (e.g., Arbuckle et al., 2016). We highlight vitamin C, or ascorbic acid, as one such promising buffering candidate for further exploration. With respect to how vitamin C (ascorbic acid) may buffer against impacts of other exposures on excitatory/inhibitory balance postnatally, vitamin C is able to modulate both excitatory (Majewska et al., 1990; Nelson et al., 2007) and inhibitory (Calero et al., 2011) function within the brain (Moretti et al., 2017). Indeed, one study has examined co‐exposures to vitamin C and alcohol administered directly to prenatal rat cortical neurons, showing vitamin C could mitigate effects on GABAergic communication induced by alcohol alone (Naseer et al., 2010). Other research has similarly found a mitigating effect of vitamin C on prenatal lead exposure for excitation/inhibition postnatally (Nam et al., 2019, 2019b). Vitamin C may also mitigate against the effects of prenatal exposures on postnatal myelination. Vitamin C has been shown to stimulate myelination processes within the brain (Eldridge et al., 1987). Rats with concurrent exposure to ascorbic acid and lead during gestation and lactation had increased myelin‐associated proteins and oligodendrocytes in the cerebellum as opposed to rats with lead exposure alone (Nam et al., 2019, 2019a). In these ways, initial evidence suggests that vitamin C may be a powerful potential mitigator of prenatal substance exposures such as alcohol and heavy metals with respect to sensitive/critical period mechanisms. Potential buffering exposures should be explored more broadly in research moving forward to inform treatment and improve care of individuals with prenatal exposures.

## 6 CONCLUSION

We have introduced a new perspective in the biological embedding of the prenatal environment by integrating the DOHaD and sensitive/critical period frameworks to characterize early neurodevelopment. To demonstrate the utility of our approach, we have focused on how two clusters of prenatal experiences richly characterized in the DOHaD model – prenatal drug/substance exposures and prenatal nutrition – shape postnatal sensitive/critical periods via impacts on two key sensitive/critical period regulators – the brain's glutamatergic excitation/GABAergic inhibition balance and myelination. Drawing from the current animal model and human neurodevelopmental evidence, we postulate how particular prenatal experiences shape sensitive/critical period timing postnatally, including precocious and/or delayed opening and closing of these important learning periods to provide targeted hypotheses for new empirical studies. We hope that articulating this integrated framework and the new directions for future research it provides will drive deeper understanding of biological embedding phenomena in early human development.

## CONFLICT OF INTEREST STATEMENT

The authors declare no known conflicts of interest.
